# The Stability of the Induced Epigenetic Programs

**DOI:** 10.1155/2012/434529

**Published:** 2012-06-03

**Authors:** Maria J. Barrero

**Affiliations:** Center of Regenerative Medicine in Barcelona, Aiguader 88, 7th floor, 08003 Barcelona, Spain

## Abstract

For many years scientists have been attracted to the possibility of changing cell identity. In the last decades seminal discoveries have shown that it is possible to reprogram somatic cells into pluripotent cells and even to transdifferentiate one cell type into another. In view of the potential applications that generating specific cell types in the laboratory can offer for cell-based therapies, the next important questions relate to the quality of the induced cell types. Importantly, epigenetic aberrations in reprogrammed cells have been correlated with defects in differentiation. Therefore, a look at the epigenome and understanding how different regulators can shape it appear fundamental to anticipate potential therapeutic pitfalls. This paper covers these epigenetic aspects in stem cells, differentiation, and reprogramming and discusses their importance for the safety of in vitro engineered cell types.

## 1. Introduction

The genome is organized into particular chromatin structures that have specific roles both in maintaining the overall structure and in gene expression. The fundamental unit of chromatin is the nucleosome, composed of two copies each of four core histones, H2A, H2B, H3, and H4, wrapped by 146 bp of DNA. The recruitment of linker histone H1 and other structural proteins can lead to further condensation and the of higher-order structures, which play additional roles in the organization of chromosomes. Chromatin offers a physical barrier to the efficient recruitment and processivity of the RNA Polymerase II (Poll l) and thus impedes gene transcription [1].

The extent of chromatin condensation is subject to regulation. The N-terminal tails of histones are relatively accessible to enzymatic modifications such as acetylation, methylation, phosphorylation, ubiquitination, and sumoylation. Furthermore, the cytosine residues of DNA can be modified by methylation and hydroxymethylation. These modifications can influence the degree of condensation of chromatin per se or/and facilitate the recruitment of structural or effector proteins, such as remodeling complexes, that directly affect the condensation of chromatin.

Certain areas of the genome are organized into heavily condensed chromatin structures, such as centromeric regions, and offer little room for transcriptional regulation. These areas are enriched in H3K9 methylation and marked by the presence of structural proteins such as HP1 (heterochromatin protein 1), which contribute to maintain high levels of condensation that play mainly structural roles in the organization of chromosomes. However, other regions of the genome are enriched in genes that are silenced but that can be active in certain situations or in different cell types. Although the mechanisms of gene silencing might be heterogeneous and gene specific, overall these areas are occupied by the Polycomb complex and marked with H3K27me3. Genes encoding many developmental regulators are located in such regions.

Tissue specific genes and developmental regulators are thus subject to intense regulation. The mechanisms leading to transcriptional activation or repression are presumably gene specific and highly influenced by the transcription factors bound at the regulatory regions of a particular gene at a given time. Extensive genomewide studies have been pursued in an effort to correlate transcriptional competence and histone modifications. This rationale is the basis of the “histone code” that postulates that the particular combination of histone modifications present at a given genomic region acts as a code to specify gene activity [2]. However, although certain modifications are strongly correlated with transcriptional activation or repression, it is often difficult to predict from the presence of a single mark the transcriptional status of a gene and even more difficult to envision the predisposition of genes to become activated or repressed. While some silent genes can be activated by certain signals others remain permanently silent and refractory to stimulation. This property is displayed in cell-specific ways and defines both cell identity and plasticity. Certain cell types, such as stem cells, have very plastic chromatin that makes them extremely sensitive to environmental signals. As cells differentiate, particular genes become silent with a consequent loss of regulatory potential.

## 2. The Epigenetic Landmarks of ES Cells

Embryonic stem (ES) cells are derived from the inner cell mass (ICM) of the preimplantation embryo and are characterized by their ability to self-renew and to give rise to virtually any cell type of the adult organism, a property called pluripotency.

A considerable amount of effort has been devoted to identify the network of transcription factors that control these two unique properties. As a result, a core regulatory network governed by the transcription factors Oct4, Sox2, and Nanog has been identified. These three factors are able to stimulate the expression of each other and also to control self-renewal and pluripotency through different mechanisms. First, they bind to the regulatory regions of genes involved in self-renewal and stimulate their transcriptional activity. Second, they can also occupy the regulatory regions of critical genes involved in development and differentiation and presumably contribute to maintain these genes in a silenced but poised state for activation during differentiation, which constitutes the basis of pluripotency [40]. How these factors mediate these two apparently opposite functions at the two types of genes is intriguing. Accumulating evidence suggests that despite the presence of self-renewal factors in both types of genes the chromatin complexes that assemble on these genes are completely different [6, 13, 20, 21].

Embryonic stem cells can be maintained in vitro in the presence of signalling molecules such as LIF, FGF, or TGFb. While mouse ES cells are dependent on LIF, human ES cells depend on the presence of FGF, which seems to sustain a pluripotent state that resembles mouse stem cells derived from the epiblasts. Overall, despite the differences between species, all these complex and still quite unexplored signalling events converge into two main responses: (1) maintenance of very high rates of transcription of genes that belong to the pluripotency network and (2) “poising” of developmental genes.

Overall, ES cells display high rates of transcriptional activity compared to differentiated cells [15], which is presumably devoted to the maintenance of high expression of pluripotency genes. Accordingly, ES cells express high levels of general transcription factors (GTFs) and of certain complexes involved in transcriptional activation such as the ATP-remodeling BAF complex and the Mediator complex [15, 21]. Moreover, differences in the expression levels of different subunits of these complexes lead to the formation of unique complexes that differ in subunit composition and potentially in function from differentiated cells [12, 21]. The relevance of these complexes is further supported by the reported loss of self-renewal caused by the depletion of the remodelling factors Chd1 or Brg1 [15, 16] and the Mediator subunit Med12 [21] in mouse ES cells. Moreover, a recent genomewide RNAi screening revealed the involvement of the chromatin remodelling complex INO80, the Mediator complex, and TBP-associated factors (TAFs) in human ES cell biology [14]. Since some of these factors have been found to cooccupy the regulatory regions of self-renewal genes with Oct4, Sox2, or Nanog [21] it is possible that they act as cofactors that contribute to support the high levels of transcription mediated by these pluripotency transcription factors. Accordingly, Brg1, subunits of the INO80 complex, and Chd1 have also been identified as part of the Oct4 interacting network [41, 42].

ESC chromatin presents structural peculiarities compared to differentiated cells. Heterochromatin appears more relaxed, perhaps due to the fact that the proteins involved in the formation of heterochromatin such as HP1 and linker histone H1 display hyperdynamic interactions with chromatin [43]. ES cells also display unique modification patterns, referred to as bivalent domains, at the regulatory regions of developmental genes. These are characterized by the presence of large regions of H3K27me3 harboring smaller regions of H3K4me3 around the transcriptional start site. The coexistence of these two antagonistic marks has been suggested to play a role in silencing developmental genes in ES cells while keeping them poised for activation upon initiation of specific developmental pathways [7, 44]. Bivalent genes are further enriched in CpG islands that in ES cells are nonmethylated. The enzyme Tet1, which is highly expressed in ES cells, has been suggested to maintain DNA in a hypomethylated state through the hydroxymethylation of CpGs at these domains [45, 46]. However, a recent study reports that Tet1 is dispensable for maintaining pluripotency of mouse ES cells [47]. Moreover, the function of DNA hydroxymethylation still remains obscure regarding its potential roles in protection against DNA methylation, providing docking sites for given factors or as an intermediary of DNA demethylation.

Despite being transcribed at very low levels, bivalent genes have considerable levels of transcriptionally engaged RNA Polymerase II near their transcription start sites but greatly reduced levels of productive elongating Pol ll [48]. However, Pol II at these promoters is confined to extremely proximal regions relative to the transcription start site and is in a conformation that is apparently different than the one found at bona fide paused locations of actively transcribed genes [3], suggesting that Pol II is stalled at these promoters in a unique conformation that can be referred to as “poised.” Knock out of Ring1B (Table 1), the Polycomb subunit that mediates ubiquitination of histone H2A, causes the loss of ubiquitinated H2A at bivalent genes, which in turn leads to changes in Pol II conformation and the derepression of the target genes. Therefore, ubiquitination of H2A seems to play a role in restraining poised Pol II at bivalent genes [3].

The regulation and the potential role of the H3K4me3 marks at these domains remain obscure but it is likely that members of the MLL family of H3K4 methyltransferases (Table 1) play a role in mediating this modification, while its deposition might favour the recruitment of Pol II to these domains [49]. Knockdown of the newly identified MLL subunit Dpy-30 [18] does not cause self-renewal defects, but rather defects in differentiation. However, knock down of the MLL complex core subunit WDR5 in ES cells has been reported to induce differentiation and loss of self-renewal [19]. More fully described is the essential role of the Polycomb complexes in the control of these domains (Table 1). Mouse ES cells null for specific Polycomb proteins result in decreased H3K27 methylation and show aberrantly induced expression of key developmental genes [6–8]. Interestingly, bivalent domains seem to be tightly regulated by the balance of activating and repressing activities. The Polycomb complex can mediate the recruitment of the H3K4 demethylase RBP2 to the bivalent domains to maintain the proper balance of H3K4 and H3K27 methylation in mouse ES cells [11]. Similarly, the H3K4 demethylase LSD1 is recruited to bivalent domains to regulate the levels of H3K4 methylation in human ES cells [20]. Moreover, subunits from the remodelling complexes BAF and NuRD (Brg1 and Mbd3, resp.) antagonistically control nucleosome occupancy at bivalent genes, with Brg1-mediated nucleosome loss associated with gene activation, and competing nucleosome stabilization by Mbd3 associated with gene repression. Interestingly, it has been suggested that hydroxymethyl cytosines serve to recruit the Mbd3/NURD complex to these domains [13]. Overall, bivalent domains appear governed by a complex and highly dynamic equilibrium of epigenetic activators and repressors that is likely to make them extremely sensitive to differentiation signals.

How the enzymes that maintain the bivalent domains in ES cells are recruited or stabilized at these particular regions is not fully understood. In Drosophila, the Polycomb complex is able to bind to specific DNA sequences [50]. This mechanism seems not to apply to mammalian cells, but some reports highlight the possibility that the extremely conserved distribution of CpG domains in the regulatory regions of developmental genes play a role in the recruitment of the Polycomb complex [51, 52]. Moreover, Tet1 has been reported to facilitate the chromatin binding of Polycomb components likely by decreasing DNA methylation levels at CpG-rich domains [53]. It is also possible that specific transcription factors that bind the regulatory regions of bivalent genes such as Oct4, Sox2, and Nanog contribute to Polycomb recruitment or stabilization. Accordingly, Nanog and Oct4 have been described to interact with complexes involved in transcriptional repression, including Polycomb subunits [41, 54]. Also, the transcription factor JARID2 has been suggested to participate in the recruitment of the Polycomb complex PRC2 to the regulatory regions of developmental regulators in mouse ES cells [10].

## 3. Epigenetic Changes during Differentiation

The in vitro differentiation of ES cells is achieved through the removal of molecules that promote self-renewal, such as LIF or FGF, and the addition of factors that induce differentiation. These changes in culture conditions lead to downregulation of the pluripotency network and to the activation or repression of developmental genes in a germ-layer-specific fashion.

Ultimately, the physiological function of bivalent domains might be to maintain important regulatory sequences accessible to the binding of relevant transcription factors that are activated by the differentiation signals. The regulatory areas, which were accessible at the undifferentiated stage, and which are not targeted by transcription factors, “close up” during differentiation becoming further inaccessible [55]. Therefore, differentiation to one particular lineage implies the permanent and irreversible silencing of genes involved in alternative lineages. Bivalent domains tend to resolve into methylated H3K4 alone for those genes that will become activated or methylated H3K27 alone for those that will be repressed during differentiation [56, 57]. Repression might be further reinforced by the incorporation of other repressive marks such as H3K9me3 or DNA methylation [58] ensuring the permanent silencing of developmental genes. The resolution of bivalent domains requires the coordinated action of histone lysine methyltransferases and demethylases. An elegant example is the role of H3K27 demethylates UTX and Jmjd3 in the activation of Hox genes during development [17] and in neuronal commitment [59]. Both demethylases are associated with MLL complexes [60, 61], suggesting that removal of the H3K27me3 mark and maintenance of the H3K4me3 at bivalent genes that become activated during differentiation are coordinated events. Importantly, a significant number of bivalent domains can remain unresolved and new bivalent genes might appear after differentiation [56], which might have consequences for the degree of plasticity that adult cells display.

Changes in subunit composition of chromatin-related complexes might also contribute to establishing the new epigenetic landscapes of differentiated cells. Such is the case of the Cbx subunits of the Polycomb complex. During differentiation, the expression of Cbx7 is down-regulated, while Cbx2, Cbx4, and Cbx8 are induced, leading to changes in the complex composition and properties [4]. In a similar fashion, changes in the expression of histone variants might also be involved in establishing the appropriate patterns of gene expression during differentiation. As an example, histone linker variant H1.0 is induced during differentiation and specifically recruited to the regulatory regions of pluripotency and developmental genes, contributing to their repression [62].

The silencing of the genes that belong to the pluripotency network is a critical event for proper differentiation. These genes become passively down-regulated due to the absence of LIF or FGF signalling and more actively due to the action of transcriptional repressors that are induced during differentiation. As a result, several mediators of repression are recruited to these genes, such as the methyltransferase G9a that has been reported to participate in the silencing of Oct4 by mediating methylation at H3K9 and contributing to the recruitment of HP1 and establishment of DNA methylation [63, 64]. The fact that in differentiated cells the regulatory regions of different genes of the pluripotency network are marked with different combinations of repressive modifications [58] further suggests that the mechanisms and epigenetic regulators that participate in their repression are likely to be gene specific.

## 4. Walking Back the Epigenetic Road during Reprogramming

Nuclear transfer experiments [23] showed for the first time that it is possible to reverse the differentiated phenotype. More recently, it became possible to generate induced pluripotent stem cells (iPSCs) from somatic cells by overexpressing specific transcription factors, most commonly Oct4, Sox2, Klf4, and c-Myc [24]. How these transcription factors impinge on the somatic cell genome to reprogram its gene expression profile is still not clear, but the low efficiency of the process suggests that somatic cells present barriers that prevent switches in cell identity. The fact that the efficiency of reprogramming can be increased by using inhibitors of DNA methyltransferases, histone methyltransferases and deacetylases [65–67] points to a critical role of chromatin as a barrier that prevents reprogramming.

Reprogramming appears to be a gradual process in which in early stages cells acquire the ability to self-renew and downregulate cell specific programs [68]. At this stage, cells can be trapped in a partially reprogrammed state in which they self-renew and continue to depend on the expression of the transgenes. A second critical phase consists of the activation of the endogenous pluripotency network, including the genes Oct4, Sox2, and Nanog. This event allows the maintenance of pluripotency in an autonomous way and independently of the transgenes. However, this stage is reached at a low frequency likely due to the inability of the transfactors to bind and activate the regulatory regions of the endogenous pluripotency genes [68]. Although the first stages of reprogramming lead to down-regulation of the expression of cell-specific genes, the complete erasure of this transcriptional memory takes place gradually after the activation of the pluripotency network [69]. Importantly, bivalent domains need to be re-established at critical developmental genes. Failure to regain this permissive status has dramatic consequences for differentiation, as found in iPSCs derived from nonhaematopoietic cells that display impaired blood-forming potential due to residual DNA methylation at loci required for differentiation into the haematopoietic lineage [70]. Moreover, the aberrant expression of bivalent genes in mouse iPSCs can be inversely correlated with their ability to give rise to viable animals by tetraploid complementation [71].

**Figure 1:**
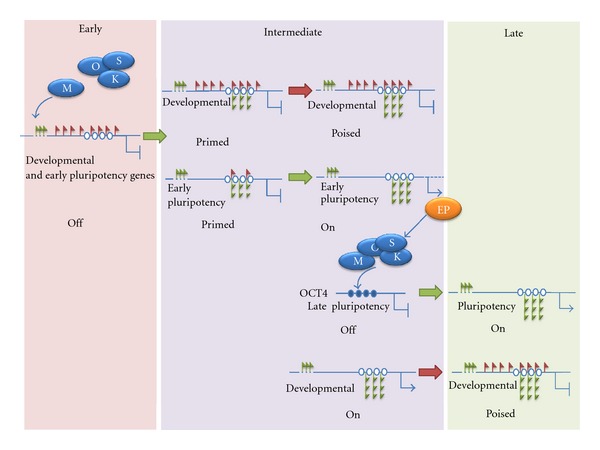
Epigenetic changes during reprogramming in genes containing CpG islands. Pluripotency and developmental genes have high CpG content and suffer dramatic changes during reprogramming. The reprogramming factors (OSKM, Oct4, Sox2, Klf4, and c-Myc) target preferentially the permissive enhancers of Polycomb target genes that are devoid of DNA methylation. These include silent developmental genes and perhaps pluripotency genes that respond early to the transfactors. As a result these genes gain H3K4 methylation at proximal promoters and are primed to become poised (developmental genes) or active (pluripotency genes) at latter stages. The products of early pluripotency (EP) genes might contribute to activate, together with the transfactors, late pluripotency genes marked with DNA methylation, such as Oct4. Finally, developmental genes become poised by gain of H3K27 methylation. Permissive enhancers are represented as dotted lines. Red flags denote H3K27me3. Green flags denote H3K4me1 (one flag), H3K4me2 (two flags), and H3K4me3 (three flags). Circles correspond to unmethylated (open) or methylated (filled) CpG islands.

Two recent studies suggest that reprogramming factors first target regions of the genome that are in a permissive chromatin conformation in somatic cells (Figure 1). Koche et al. [72] analyzed the expression patterns and epigenetic landscapes of fibroblasts at very early stages of reprogramming. At this phase, changes in gene expression seem limited to down-regulation of the somatic specific program, even though epigenetic changes can be readily detected at promoters that are already in an open and accessible conformation. Most conspicuous early event consists of the gain of H3K4me2 at promoters that are typically marked with H3K4me3 in ES cells, such as certain pluripotency and early developmental genes. These changes are restricted to sites of high CpG density, which are devoid of DNA methylation both in fibroblasts and ES cells, and in which reprogramming factor regulatory motifs might be present. Using a different approach, Taberlay et al. [73] describe that developmental genes marked with H3K27me3 at their promoters contain permissive enhancers that are depleted of nucleosomes and marked with H3K4me1 in somatic cells. These enhancers are likely to be targeted by the reprogramming factors at initial stages of reprogramming.

In contrast, the gain of DNA hypermethylation typical of ES cells and the reestablishment of H3K27me3 at bivalent promoters take place late in the reprogramming process [72]. Therefore, the acquisition of the facultative heterochromatin typical of ES cells might be a late critical step that cells need to overcome during reprogramming. Indeed the silencing of tissue specific genes appears to be more important than previously thought for the process of reprogramming, as suggested by the discovery that somatic cells can be fully reprogrammed to pluripotency by overexpression of the miR-302/367 cluster alone [36].

## 5. Discordances between ES and IPS Cells

The process of somatic cell reprogramming generates cells with similar properties to ES cells but an important question still remains: how similar are iPS and ES cells? Studies looking at global gene expression and the epigenome suggest that they are in fact quite similar and that iPS cells are unequivocally different from the somatic cells of origin [74, 75]. However, particular differences have been identified whose significance remains to be determined [37, 76]. The comparison between ES and iPS cells is not straightforward for two main reasons: (1) ES cells themselves show variability between lines; (2) the different strategies to generate iPS cells and culture techniques used in different laboratories make it difficult to tease out as to which differences are due to variations in experimental procedures and which are intrinsic to iPS cells.

Reflecting the gradual nature of the reprogramming process, early passage iPS cells retain residual expression of genes from the cell of origin, which has been proposed to facilitate their differentiation back into those very same cell types [69, 77]. However, the expression of these genes tends to disappear at late passages [69, 78]. Importantly, it has been described that a certain number of genes are still differentially expressed in several lines of iPS cells at late passages [76], although the consequences of this differential expression still need to be addressed.

More important is perhaps the potential differences in the epigenomes of iPS and ES cells, since this is likely to influence the ability of cells to differentiate and the quality of the final differentiated products. Regarding DNA methylation at CpGs, genome-wide analyses revealed that a significant number of developmental genes retained significant levels of DNA methylation at their regulatory regions in early passage iPS cells [79]. However, a very similar methylation profile between ES cells and iPSC was found in these genes at late passages [37]. Overall, iPS cells show a few hundred differentially methylated regions when compared to ES cells, corresponding both to somatic memory and to aberrant methylation [37, 79]. Different iPS cell lines share only a small number of differentially methylated regions, suggesting that there is significant reprogramming variability with regard to DNA methylation. However, some hot spots of shared differential methylation between lines have been found [37, 76]. More specifically, defective re-establishment of DNA methylation at particular loci that correlates with sustained expression of a few somatic genes has been reported [76]. Regarding non-CpG DNA methylation, iPS cells show hypomethylation in large regions proximal to centromeres and telomeres compared to ES cells [37]. Since the role of non-CpG DNA methylation is not clear, it is difficult to predict the potential outcome of this difference. A recent comprehensive study [74] combined analysis of global gene expression and DNA methylation in undifferentiated and differentiated cells to score for the ability of human ES and iPS cells to differentiate into certain lineages. The authors concluded that no common distinctive pattern was shared by all iPS cell analyzed and that reprogrammed cells were not functionally distinguishable from ES cells.

## 6. Future Goal: Assess the Epigenetic Stability to Ensure Safety

The prospect of potential uses of iPS cells in autologous therapies made researchers rush into the development of nonintegrative approaches to deliver the lowest number of factors into somatic cells [80] (Table 2). However, the idea that the absence of viral integrations would make these cells safe for therapy appears naïve if we take into account two main facts. First, iPS cells are expected to have the same drawbacks of ES cells, that is, the generation of differentiated products that are plastic enough to integrate into the damaged tissues but differentiated enough to avoid the formation of tumors. Second, iPS cells not only display epigenetic aberrancies but they also have accumulated a number of genetic mutations during reprogramming and expansion in culture [38, 39].

Laboratories around the world have now started to generate therapeutic cell types by transdifferentiation, such as blood, neurons, or cardiomyocytes from fibroblasts [33, 81, 82]. Assessing the similarity of these cells or the ones obtained from pluripotent cells to their in vivo counterparts is not straightforward. Moreover, it is likely that these cells obtained in vitro differ significantly from those found in the body. But perhaps more important is to understand the potential adverse affects that these cells can cause. Important questions emerge. How stable are these newly induced programs? What is the probability that the transplanted cells revert to less differentiated and proliferative phenotypes? Importantly, transdifferentiation is often driven by the action of master transcriptional regulators or pioneer transcription factors able to induce whole new programs of gene expression. These master genes, which might be also regulated during early stages of reprogramming [73], are targets of Polycomb and are enriched in CpG islands that are usually DNA hypomethylated in normal tissues but become often hypermethylated in cancer [79, 83] and during aging [84]. This suggests that regions of the genome that are subject to intense regulation and that play important roles in defining cell identity might be also more likely to suffer pathological deregulation.

The answer to the above questions unequivocally lies in the epigenome and how the regulators of the epigenetic marks can secure the stability of the newly established epigenetic programs. A better characterization of the epigenetic landscape, including the identification of critical genomic areas with tendency to suffer aberrant or unstable epigenetic reprogramming and the activities involved in their regulation, will be needed in order to predict the safety of the induced cell types.

## Figures and Tables

**Table 1:** Chromatin- and transcription- related complexes involved in maintaining ES cells pluripotency. The main reported activity of the complex is indicated; however, notice that specific subunits might carry out enzymatic activities different than the main described activity.

Complex	Main activity	Subunit	Reported function
Polycomb			
PRC1	H2AK119 ubiquitination	Ring1B	Restrains Pol II at bivalent genes avoiding premature differentiation [3]
		CBX7	Maintenance of pluripotency regulating PRC1 targets [4]
PRC2	H3K27 methylation	Ezh2/1	Repression of differentiation genes [5]
		Eed	Repression of differentiation genes [5–7]
		Suz12	Repression of differentiation genes and needed during differentiation [8, 9]
		JARID2	Recruitment of Polycomb to target genes [10]
		RBP2	Mediates H3K4me demethylation at bivalent genes [11]
BAF	Nucleosome remodelling	Brg1	Coactivator of the pluripotency network [12]
NuRD	Nucleosome remodelling	Mbd3	Nucleosome stabilization at bivalent domains [13]
INO80	Nucleosome remodelling	INO80	Co-activator of the pluripotency network [14]
CHD1	Nucleosome remodelling	Chd1	Co-activator of the pluripotency network [15, 16]
MLL	H3K4 methylation	UTX/jmjd3	H3K27me demethylation of bivalent domains [17]
		Dpy-30	Participates in the induction of developmental genes during differentiation [18]
		WDR5	Co-activator of the pluripotency network [19]
CoREST	Histone deacetylase	LSD1	H3K4me demethylation of bivalent domains [20]
Mediator	Transcription activation	Med12	Co-activator of the pluripotency network [21]

**Table 2:** Main breakthroughs regarding reprogramming and transdifferentiation of somatic cells. The need of oocytes and the low efficiency of nuclear transfer in humans have propitiated the search for alternative strategies to generate pluripotent cells. Induced pluripotent cells, initially obtained with retroviruses encoding Oct4, Sox2, Klf4 and c-Myc (OSKM), were considered unsafe for therapy due to the presence of viral integrations and the use of oncogenes Klf4 and c-Myc. Therefore, a major rush to develop non integrative methods and to avoid the use of oncogenes started. However, the finding that iPS cells have epigenetic and genetic aberrations suggests that these cells will need to be analyzed in detail before moving to the clinic.

Year	Breakthrough
1987	Fibroblast transdifferentiation to muscle cells [22]
1997	Pluripotent cells by nuclear transfer [23]
2006	Mouse iPS cells with OSKM retroviruses [24]
2007	Human iPS cells with OSKM retroviruses [25]
2008	IPS cells without c-Myc [26]
	iPS cells from neural stem cells with two factors [27]
	iPS cells with two factors and small molecules [28]
	iPS cells with non integrative viruses [29]
	Desease-specific iPS cells [30]
2009	iPS cells with proteins [31]
2010	iPS cells with RNA [32]
	Transdifferentiation of fibroblasts to neurons or to cardiomyocytes [33, 34]
	Transdifferentiation of fibroblasts to blood cells [35]
2011	iPS cells with miRNAs [36]
	iPS cells have epigenetic aberrations [37]
	iPS cells have genetic aberrations [38, 39]
